# The Strong Selective Sweep Candidate Gene *ADRA2C* Does Not Explain Domestication Related Changes In The Stress Response Of Chickens

**DOI:** 10.1371/journal.pone.0103218

**Published:** 2014-08-11

**Authors:** Magnus Elfwing, Amir Fallahshahroudi, Isa Lindgren, Per Jensen, Jordi Altimiras

**Affiliations:** AVIAN Behavioural Genomics and Physiology Group, Division of Biology, IFM Biology, Linköping University, Linköping, Sweden; CSIRO, Australia

## Abstract

Analysis of selective sweeps to pinpoint causative genomic regions involved in chicken domestication has revealed a strong selective sweep on chromosome 4 in layer chickens. The autoregulatory α-adrenergic receptor 2C (*ADRA2C*) gene is the closest to the selective sweep and was proposed as an important gene in the domestication of layer chickens. The *ADRA2C* promoter region was also hypermethylated in comparison to the non-selected ancestor of all domesticated chicken breeds, the Red Junglefowl, further supporting its relevance. In mice the receptor is involved in the fight-or-flight response as it modulates epinephrine release from the adrenals. To investigate the involvement of *ADRA2C* in chicken domestication, we measured gene expression in the adrenals and radiolabeled receptor ligand in three brain regions comparing the domestic White Leghorn strain with the wild ancestor Red Junglefowl. In adrenals *ADRA2C* was twofold greater expressed than the related receptor gene *ADRA2A*, indicating that ADRA2C is the predominant modulator of epinephrine release but no strain differences were measured. In hypothalamus and amygdala, regions associated with the stress response, and in striatum, receptor binding pIC50 values ranged between 8.1–8.4, and the level was not influenced by the genotyped allele. Because chicken strains differ in morphology, physiology and behavior, differences attributed to a single gene may be lost in the noise caused by the heterogeneous genetic background. Therefore an F_10_ advanced intercross strain between White Leghorn and Red Junglefowl was used to investigate effects of *ADRA2C* alleles on fear related behaviors and fecundity. We did not find compelling genotype effects in open field, tonic immobility, aerial predator, associative learning or fecundity. Therefore we conclude that *ADRA2C* is probably not involved in the domestication of the stress response in chicken, and the strong selective sweep is probably caused by selection of some unknown genetic element in the vicinity of the gene.

## Introduction

Domestication is a swift evolution of traits propelled by human-driven selection [1]. For most domestic species a strong selection to adapt to a man-made environment has generated a colorful palette of morphological, physiological and behavioral novel phenotypes. The chicken is a prime example of domestication. Strong artificial selection on somatic growth and egg production has produced chickens more than five times larger (at the same age) or with higher fertility (more than twice the number of eggs laid) than the original Red Junglefowl ancestor [2]. Not only production traits but also behavioral traits such as fearfulness, stress resilience, social tolerance, and morphological traits such as feather and leg coloration, feather structure and sexual ornaments have changed remarkably [3]–[6]. Indeed, one of the main traits under selection, at least early in domestication, was stress coping behavior and reduced stress response [7]. Domestic chickens possess lower stress responses in open field, aerial predator, novel object and fear of human tests demonstrated by lower frequencies of immobilization and freezing, and higher frequencies in vocalization, walking and flying [4].

To characterize domestication at the genetic level several studies have used quantitative trait loci analysis (QTL) and whole-genome resequencing and selective sweep analysis comparing the wild ancestor to domestic breeds [8]–[11].

Selective sweep analysis is a method to identify positively selected gene variants important for domestication. A sweep region is characterized by loss of genetic variation indicating strong positive selection and is assumed to be associated with a mutation causing desirable traits. Among the domestication related selective sweeps identified by Rubin et al. (2010), one of the strongest ones is located adjacent to the coding region for α-adrenergic receptor 2C (*ADRA2C*) [12]. The sweep is 120 kb long on chromosome 4, and in a region void of known genes except for *ADRA2C* which is situated 10.8 kb outside of the region. Hence, *ADRA2C* stands out as the most likely candidate causing the sweep.

ADRA2C is predominantly present in the central nervous system and in the adrenals [13]. ADRA2C works as an autoreceptor on presynaptic sympathetic neurons and it is involved in the regulation of norepinephrine release by a negative feedback loop. It is also the main regulator of epinephrine release from the adrenal medulla as shown in *Adra2C* knockout mice showing more than a 2-fold increase in circulating epinephrine [14]. Behavioral alterations in *Adra2c* knockout mice are seen as an enhanced startle response, shortened attack latency and diminished acoustic prepulse inhibition [15]. These findings were reversed in mice overexpressing *Adra2C*. For these reasons ADRA2C is tightly linked to the fight-and flight response and is a strong candidate gene to explain the effects of domestication on stress related behaviors.

*ADRA2C* is also subjected to epigenetic modulation and the domestic phenotype in White leghorn (WL) is by being significantly hypermethylated compared to wild type Red Junglefowl (RJF) [16]. Cysteine methylation on CpG-islands modulates gene expression and can be inherited [17] suggesting that domestication is not limited to only selection on genetic variance but could also operate on epigenetic signatures.

We hypothesized that the selective sweep on chromosome 4 adjacent to the *ADRA2C* locus is associated with regulatory effects on *ADRA2C* expression, which could affect behavior in stressful situations. Gene expression and receptor density was evaluated in the adrenals and in the brain in RJF and WL chickens. Because these strains differ in behavior and physiology, and because of the difficulties associating a behavioral difference with a candidate gene between heterogeneous groups we used an F_10_ advanced intercross line between RJF and WL homozygous for alternative *ADRA2C* alleles for behavior and fecundity studies. Using this method, we could study the effect of the domestic genotype compared to a mosaic genomic background that has resulted from 9 generations of accumulating recombination between the two chicken strains.

## Materials And Methods

### Bioinformatics

**Figure 1:**
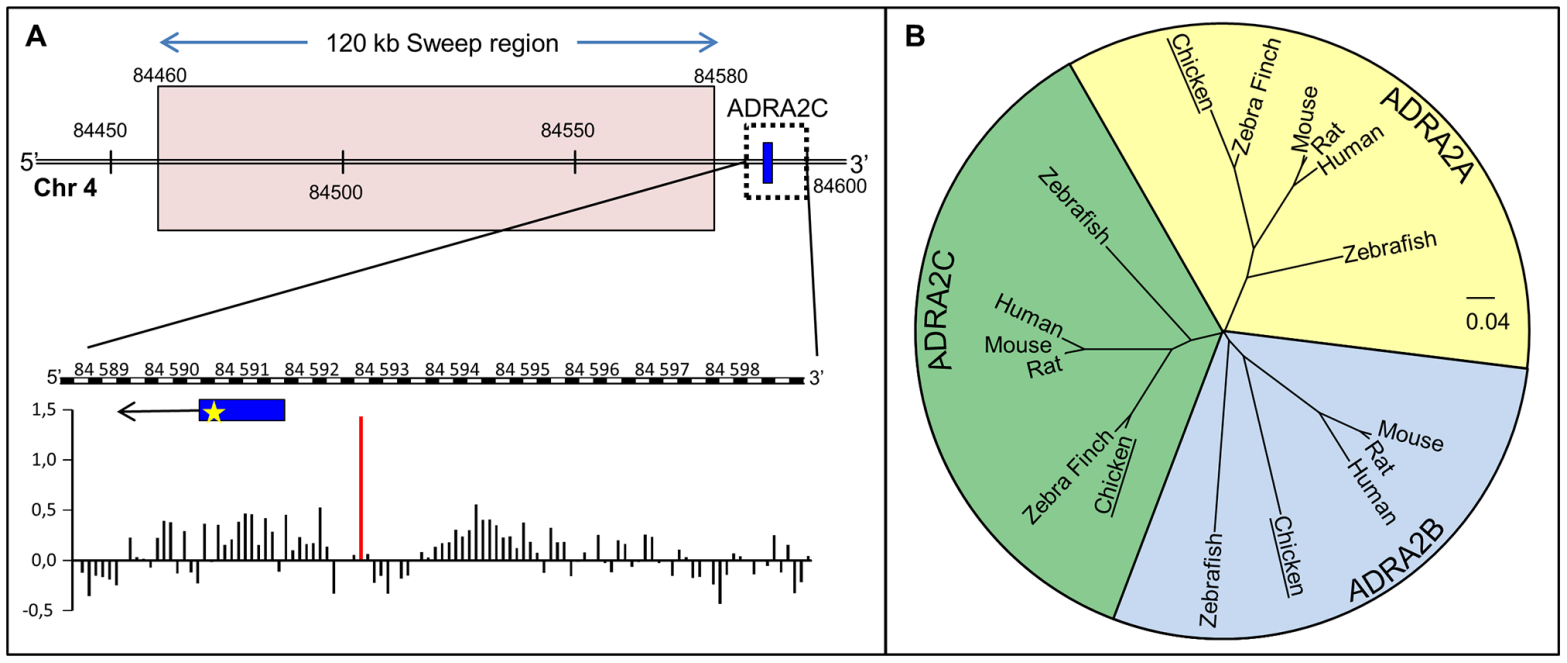
Positional information on the location of ADRA2C in relation to a neighboring selective sweep and methylated regions from previous studies. A) Location of the *ADRA2C* in chromosome 4 in relation to the selective sweep region reported by Rubin et al. (2010). All coordinates are expressed as kb. The position of the 1229 bp long gene starting at 84590769 is represented by a blue box and the SNP in position 84590997 used for genotyping is shown as a yellow star within the gene. The direction of transcription is indicated with an arrow. The methylation pattern bp long gene starting at 84590769 is represented by a blue box and the SNP in position 84590997 used for genotyping is shown as a yellow star within the gene. The direction of transcription is indicated with an arrow. The methylation pattern (in Log2 fold change) comparing RJF and WL, shown with a resolution of 50–75 bp regions is shown in the bar graph, and the hypermethylated region is highlighted with a red bar at position 84593087. Methylation data from N bp regions is shown in the bar graph, and the hypermethylated region is highlighted with a red bar at position 84593087. Methylation data from Nätt et al. [16]. B) Peptide sequence homology phylogeny of the annotated sequences of α_2_ adrenergic receptor subtypes cluster the chicken *ADRA2C* gene to better characterized mammalian ADRA2C genes.

The position of the 120 kb long selective sweep window stretches between chr4:84.46–84.58 mb [12] in ENSEMBL WASHUC2 database. *ADRA2C* consists of a single 1229 bp long exon starting at chr4:84590769 transcribed 3′ to 5′ and situated 10 769 bp from the selective sweep. The significant hypermethylation in the presumed promoter region consists of a differentially methylated 50 bp-region located at chr4:84593087, a pattern seen regularly as an epigenetic modification [16] (Figure 1A).

Peptide sequences of the three alpha-adrenergic receptor subtypes from 6 species (chicken, Zebra Finch, mouse, rat, human and zebrafish) were used to create a multiple sequence alignment and a neighbor joining phylogenetic tree using Clustal 2W. All genomic coordinates used to generate the tree originate from WASHUC2/galgal3 and can be found in Table S1.

### Animal Care And Handling

The experiments were approved by the “Regional Committee for Ethical Approval of Animal Experiments” in Linköping, Sweden (permit no 122-10). Three different chicken strains were used in this study: captive RJF, WL and a 10^th^ generation intercross between them (F_10_). RJF is the wild ancestor to all domestic chicken breeds and our captive population originates from Thailand and has been maintained in captivity for around 15 years. WL strain (SLU13) has originally been strongly selected for egg production and stems from an outbred mixture of breeds established 1970. Since then it has been maintained as a closed population at the university. The detailed history of these populations has been described elsewhere [2],[3].

RJF and WL are known to differ in behavior as well as growth and fecundity [2],[3], and for these reasons they would suit poorly as models to investigate the impact of a specific gene. Instead, F_10_ birds of an advanced intercross were used for behavioral studies. Details regarding the origin, management and characteristics of the intercross line have been explained in detail elsewhere [2],[18]. In short, one RJF male was paired with three WL females to generate the F_1_ population. Four F_1_ males and 36 F_1_ females founded the F_2_ generation and a new generation has been bred approximately every 1.5 year through pedigree hatching. The population for this study was obtained from parental F_9_-birds which had been genotyped for the *ADRA2C* locus on chromosome 4 and 14 heterozygote families yielded 168 pedigree hatched offspring in two batches. These birds showed a Mendelian inheritance on the *ADRA2C* locus where 78 birds were heterozygote (A^+^/A^D^), 43 were homozygote for the RJF wild type allele (A^+^/A^+^) and 44 homozygotes for the WL domestic allele (A^D^/A^D^). Out of these birds, 136 were used for behavioral studies as follows: 56 A^+^/A^D^ (17 males, 39 females), 40 A^+^/A^+^ (16 males, 24 females), and 40 A^D^/A^D^ (18 males, 22 females).

Detailed rearing and breeding routines are reported in previous publications from our group [4]. Briefly, all animals were kept separated by strain, but not sex or genotype, at Linköping University poultry facilities with food and water available *ad libitum* held under controlled temperature and light regimes (12∶12 light/dark cycle, 5 lux) in 1–2 m^2^ pens depending on age for their first seven weeks.

### Somatic Growth, Fecundity And Genotyping

The F_10_ population was used for growth, fecundity and behavioral studies, and for genotyping purposes 75 µl of blood was collected from the left ulnar vein in a capillary tube. DNA was extracted using DNeasy extraction kit from Qiagen according to the instructions from the manufacturer. The animals were genotyped using a synonymous single nucleotide polymorphism (SNP) in the *ADRA2C* gene positioned on chromosome 4:84590997 functioning as a proxy for the selective sweep. A 320 bp long product was amplified with touchdown qPCR (CG Palmcycler, Genetix Biotech) with a 3 min, 95°C initiation step followed by a 30 s 95°C melting, 30 s 62–53°C annealing and 1 min 72°C elongation step in 45 cycles. Streptavidin Sepharose High Performance base matrix (GE Healthcare Life Sciences) was used to bind the biotin labeled reverse primer. The sequence of all primers used could be found in Table S2. PyroMark reagents were used following the instructions from the manufacturer using a Pyromark Q24 work station (Qiagen).

To assess somatic growth in the F_10_ population, body mass and length of the tarsometatarsus were measured weekly for the first seven weeks starting at the day of hatching. Adult body mass and tarsometatarsal length were obtained at 88 days.

For the measurement of fecundity of the F_10_ advanced intercross females were moved to 40×100×50 cm isolation cages at 24 weeks of age to habituate 4 d prior to the initiation of egg sampling. They were in visual and vocal contact with each other, held on a 12∶12 diurnal rhythm at 20°C room temperature with *ad libitum* access to food and fresh water. Eggs were collected and weighed daily during a two week period.

### Behavioral Tests

#### Open Field

To assess anxiety and exploration related behavior the animals were tested in a novel open field arena. At four weeks of age the bird was placed in darkness in a 80×120×40 cm novel arena. The arena was divided in three virtual zones, start zone, center zone (40×80 cm) and peripheral zone and total movement, latency of leaving start zone, velocity and time spent in each zone (start zone was included in peripheral zone for this measurement) was measured for 10 minutes using ceiling-mounted video cameras and the automatic tracking software Ethovision (Noldus, version 3.1).

#### Associative Learning

At 5 weeks of age all chicks were subjected to an associative learning test. The task was to discriminate between two colors, blue and yellow, associated with a mealworm as reward. 80×120×40 cm arenas were used with two feed cups, one containing a mealworm, balanced for color (blue and yellow) and spatial position (right and left) to avoid color preference and spatial bias. All cups contained wood shavings to avoid visual detection of the worms.

The animals were allowed to habituate in the arena for 10 min allowing them to detect the mealworms and associate color with reward. If this failed the observer placed a mealworm in front of the cup, and if this too failed a trained social companion was introduced.

Five trials during consecutive days were performed and latency of making a choice and number of correct choices were scored. If the animal had not made a choice within 10 minutes, the trial was ended, and if the bird did not make a choice in two consecutive trials the bird was excluded. Trial one, two and four were performed as described above, trial three was carried out without a reward to discard the effect of smell, vision or hearing on preference, and in trial five the position of the cups was swapped to account for spatial learning.

#### Tonic Immobility

Tonic immobility (TI, a catatonic state lasting for up to several minutes) is a replicable and valid measure of fear reactions in chickens [19]. Animals were tested blindly at 7 weeks of age as reported earlier [20] with a few modifications. In short the birds were placed on their backs in a V-shaped wooden cradle gently applying a force on their chest to induce TI. If the animal did not stay in TI for more than 10 seconds, the attempt was considered unsuccessful and the induction attempt was repeated (no more than 5 times). After successful induction, numbers of attempts and total time duration to first head movement and latency of righting was recorded. If the bird remained in TI after 600 s the bird was assigned the maximal value, and after completion of the test the bird was returned to its home pen.

#### Aerial Predator

The response to a simulated aerial predator assesses fear reaction and serves as a measure of the fight-and-flight response. At 14 weeks of life the animals were exposed to an artificial aerial predator as previously reported [21]. In short, the bird was placed, in darkness, in a 50×150×50 cm test arena and was allowed to habituate for 2 min. The animals were observed with direct recording by an observer hidden by a screen using one-zero sampling at 10 s intervals. The test comprised a pre-exposure period of 5 min functioning as baseline, an exposure of a hawk model (wingspan 62 cm, length 31 cm) attached to a pulley slid above the bird with a presentation time of 2 s, and a 5 min post-exposure period. Eight different behaviors, and vocalization were recorded and behaviors were classified as relaxed (exploring, pecking, preening, sitting) or agitated (stand alert, walk alert, freezing and attempting to escape) and a ratio was calculated as performed behavior divided by the total data points.

### Molecular Studies

For gene expression and receptor binding studies 3 week old RJF and WL chickens were used. All animals were genotyped to verify the correct homozygous genotype, using the marker and primers described above.

#### Gene Expression

For gene expression, chickens were euthanized by decapitation and the hypothalamus enriched region was anatomically dissected out (3 female and 2 male RJF, 4 female and 2 male WL), and adrenals (5 females and 6 males RJF, 10 females and 8 males WL) were harvested, within 8 minutes, frozen in liquid nitrogen and stored in −80°C until processing. Total RNA was isolated using Ambion TRI reagent (Applied Biosystems, Carlsbad, CA, USA) in tubes containing ceramic beads (Lysing matrix D, Biomedicals) and a Fastprep-24 homogenizer (MP Biomedicals, Solo, OH, USA) following the instructions from the manufacturer. Single stranded cDNA for qPCR was synthesized using Fermentas (St. Leon-Rot) RevertAid Reverse Transcriptase, 10 nM dNTPs, Ribolock nuclease inhibitor and Oligo(dT)_18_ primer (Thermo Fisher Scientific, Freemont, CA, USA) according to the instructions from the manufacturer. qPCR was performed with a LightCycler 480 (Roche Applied Science) using Maxima SYBR Green qPCR mastermix (Thermo Fischer Scientific) in 10 µl reactions. The qPCR protocol consisted of a 5 min 95°C activation step followed by 45 cycles of 10 s 95°C melting, 10 s 55°C annealing and a 20 s 72°C elongation and finally the product was melted with increasing temperature from 72°C to 95°C. The relative gene expression was quantified using the 2^−ΔΔ*C*^_T_ method [22] normalized to TATA box binding protein and RNA polymerase II subunit C1. β*2* microglobulin was used as a third housekeeping gene for gene expression measurements in the hypothalamus. All primer sequences are reported in Table S2. Two samples were included to all plates as inter-plate calibrators.

#### Receptor Binding

For receptor binding purposes WL and RJF chicks (5 females and 4 males RJF, 5 females and 4 males WL) were euthanized by decapitation. The brain was rapidly removed (within 6 min) and snap-frozen in isopentane cooled by liquid nitrogen and stored in −80°C until processing (within 4 months). On the day of processing the tissue was embedded in cryo-medium (Tissue Tek O.C.T compound, Sakura) and cut with a cryostat (Microm HM 550, cellab) in 350 µm coronal sections from the frontal telencephalon to the cerebellum. The sections were placed on glass slides and covered with 50 microliters of phosphate buffered saline (PBS). The amygdala, the hypothalamus and the striatum were dissected out from the tissue slices under a microscope. The tissue was homogenized and diluted with PBS to a final volume of 3.2 ml and kept on ice. Tritium-labelled Rauwolcin (PerkinElmer) to a final concentration of 2 nM together with the ADRA2C specific ligand Spiroxatrine (10^−11^, 10^−10^, 10^−9^, 3×10^−9^, 10^−8^, 3×10^−8^, 10^−7^ and 10^−6^ M) was added to 176 µl homogenate in triplicates and incubated on ice for 2 h. Duplicated homogenate aliquots were used for protein analysis using a standard protein analysis kit (Pierce BCA Protein Assay Kit; Thermo Fisher Scientific) following the instructions from the manufacturer. The homogenates were then transferred to a Multiscreen Filter plate (Millipore). Vacuum was applied to the multiwell manifold using a water aspirator and the homogenate was filtered through. All wells were washed 3 times with PBS. The glass fiber filters were punched out from the filter plate and placed in 7 ml Scintillation vials filled with 3 ml Scintillation cocktail (Optiphase ‘Hisafe’ 3, PerkinElmer) and incubated overnight. ^3^H decay was measured in a liquid scintillation counter (LS 6500, Beckman Instruments, Fullerton, CA).

### Statistical Analysis

For all pairwise comparisons with n-values lower than 30 a permutation test was performed using the Statboss Permutation tester 1.0 (http://www.pharmacology.unimelb.edu.au/statboss/home.html) [23]. For comparisons with n-values higher than 30 a Student's t-test was performed. All values are expressed as average and standard deviation.

## Results

A peptide sequence homology phylogeny was constructed from the annotated sequences of α_2_ adrenergic receptor subtypes in different species (Figure 1B). The chicken ADRA2C clusters together with the same receptor subtype in all species included in the comparison confirming the receptor subtype on the basis of sequence homology. α_2A-_ and α_2B_-adrenergic receptors also showed a larger intrinsic homology between species than with any other subtype.

Differences in *ADRA2C* genotypes did not affect growth of the advanced intercross birds at any age (Table S3). There was an expected difference between sexes from 8 d of development onwards with males being approximately 30% heavier after 88 days (Table S3). The length of the metatarsus, as a measurement of somatic growth, showed no significant difference between genotypes at any age, but males exhibit longer metatarsus with age compared to females (data not shown).

**Figure 2:**
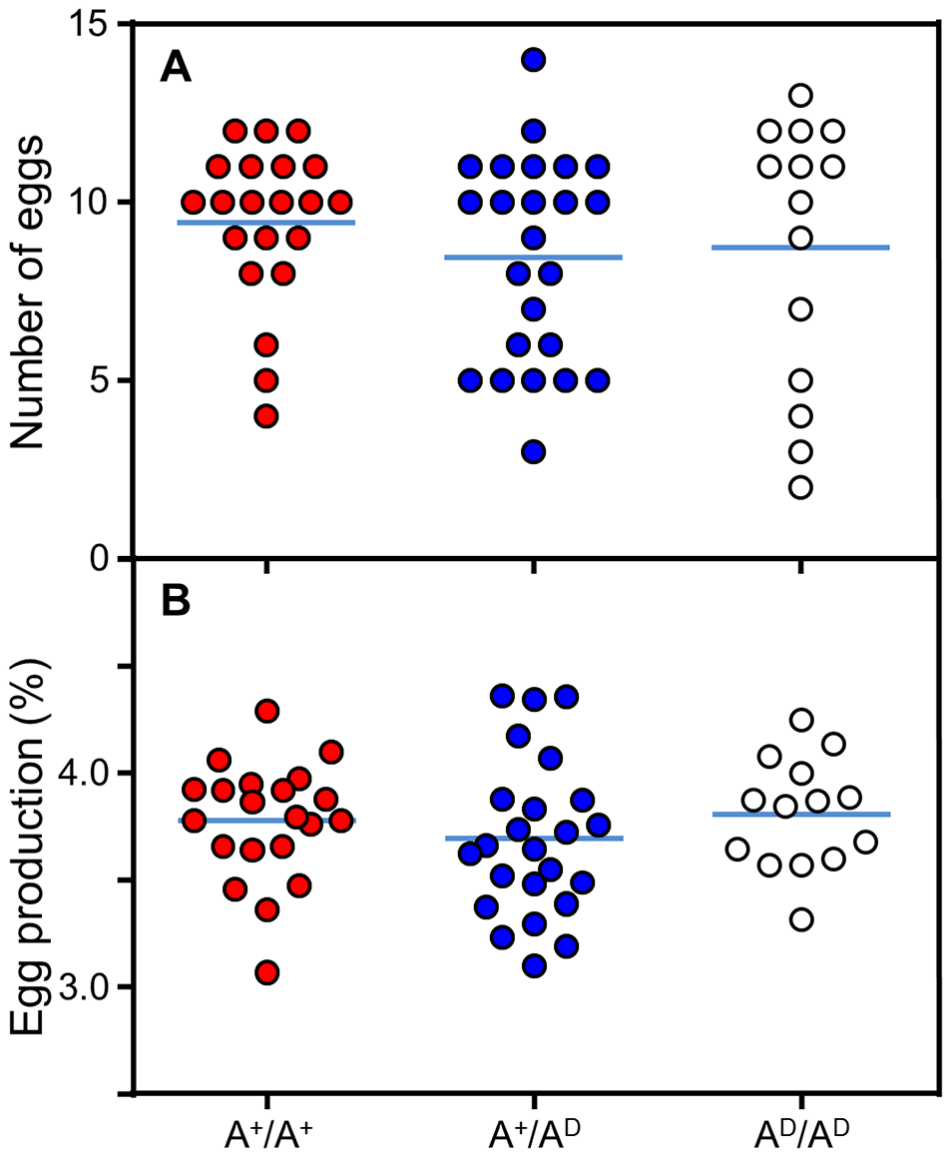
Fecundity results from the different genotypes of the F_10_ intercross strain. A) total egg count during the 2 week collecting period and B) total egg mass as a percent of body mass. A^+^/A^+^ is wild type, A^+^/A^D^ is heterozygote and A^D^/A^D^ is domestic genotype. The horizontal line shows the mean value and the dots are the individual data points.

No genotype dependent differences in fecundity were observed. On average 0.6 eggs were laid daily weighing 3.7% of the hens' body mass independently of genotype (Figure 2).

**Figure 3:**
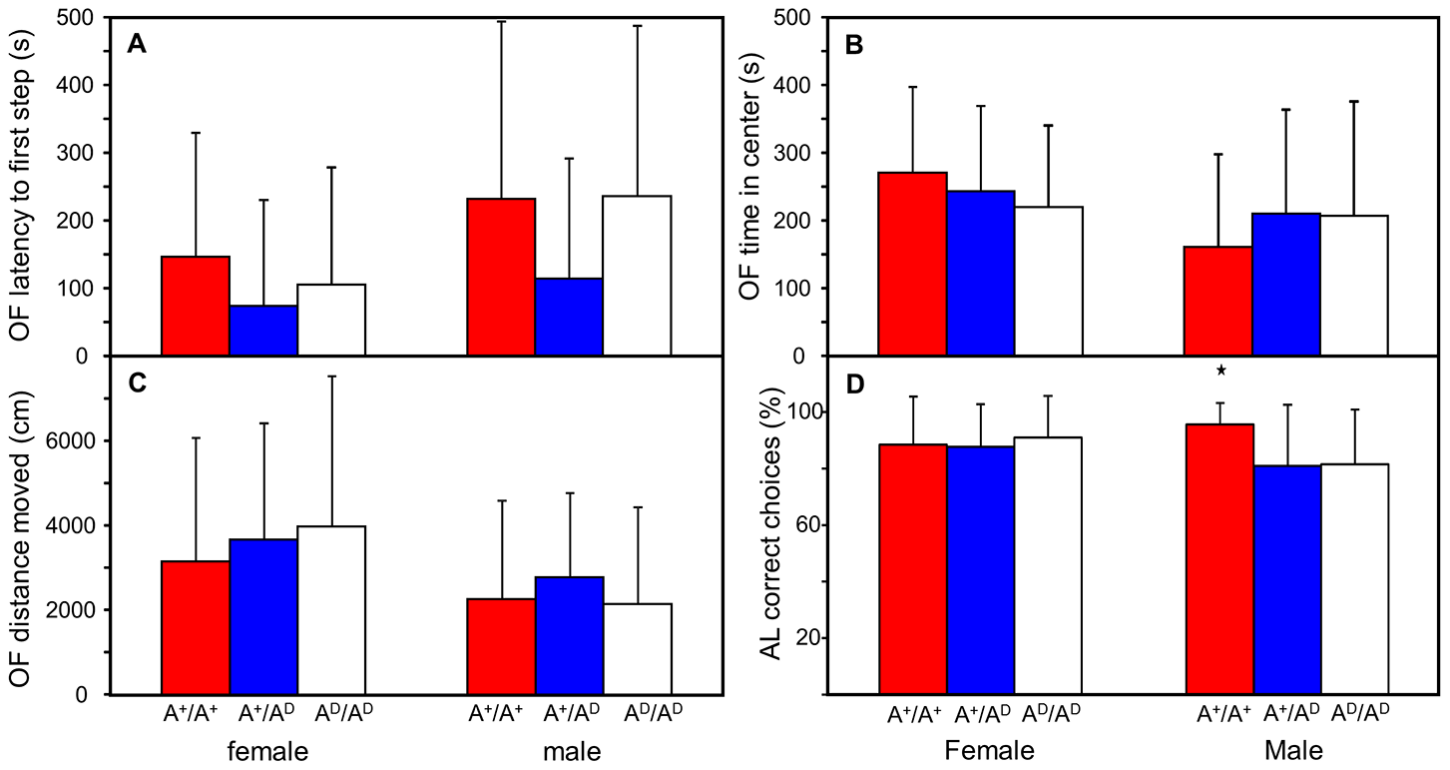
Behavioral variables in Open Field (OF) and Associative Learning (AL) tests. A) Latency (s) to first step in OF test. B) Total time spent in the center of the arena (s) in OF test. C) Distance moved (cm) in OF test. D)) Correct choices (%) in AL test. Data shown as mean and standard deviation.

Behavior in open field tests varied significantly between sexes so all behavioral data is presented separately for each sex. No significant differences were found between genotypes for any of the Open Field variables (Figure 3A–C).

In an Associative learning test females showed a success rate of approximately 90% without influence of genotype (Figure 3D). In males however, A^+^/A^+^ animals succeeded significantly better than A^+^/A^D^ and A^D^/A^D^ animals with 95% success in comparison to 80% in the other groups (P = 0.017 between A^+^/A^+^ and A^D^/A^D^, and 0.018 between A^+^/A^+^ and A^+^/A^D^).

**Figure 4:**
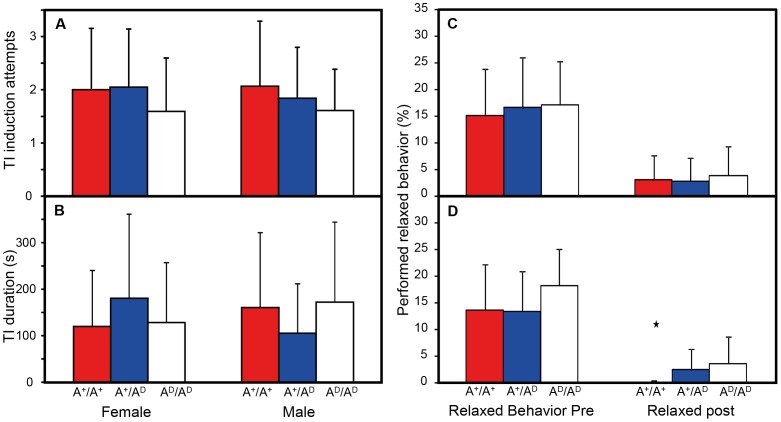
Behavioural variables from Tonic Immobility (TI) and Aerial Predator tests. A) TI induction attempts and B) TI duration (s). C) and D) Relaxed behavior pre and post exposure to the aerial predator in males (C) and in females (D). Data shown as mean and standard deviation. Star indicates a significant difference between genotypes (p<0.05).

The number of attempts to initiate tonic immobility (1.9±1 attempts on average shown in Figure 4A) and duration (144±146 s) did not differ between genotypes or between sexes (Figure 4B).

All animals were strongly affected by the exposure to the raptor in the aerial predator test. A great reduction in the performed relaxed behavior post exposure was seen (Figure 4C–D). In females, no difference was found between genotypes, but in males, A^+^/A^+^ animals showed significantly less relaxed behaviors post exposure compared to both A^+^/A^D^ and A^D^/A^D^ birds (P = 0.005 compared to A^D^/A^D^ and P = 0,014 compared to A^+^/A^D^).

**Figure 5:**
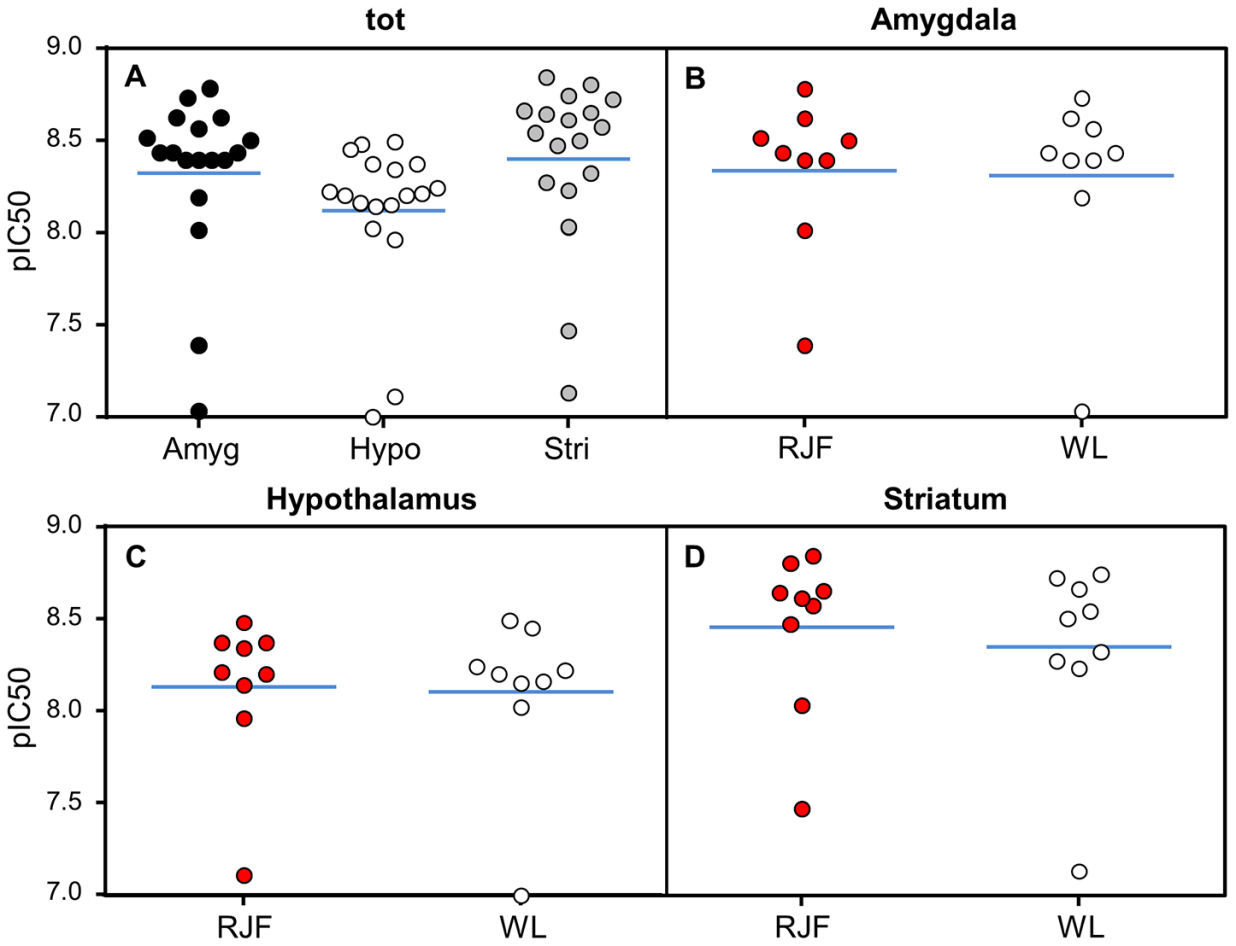
ADRA2C abundance from receptor binding assays. A) Receptor abundance in 3 brain regions with all animals pooled. Abundance in amygdala (B), hypothalamus (C) and striatum (D). The line indicate the mean value and dots indicate individual data points.

No differences were found between sexes in the receptor binding assay so data from males and females was pooled. *ADRA2C* was expressed in amygdala, hypothalamus and striatum and no differences were found between RJF and WL in either brain region (Figure 5).

**Figure 6:**
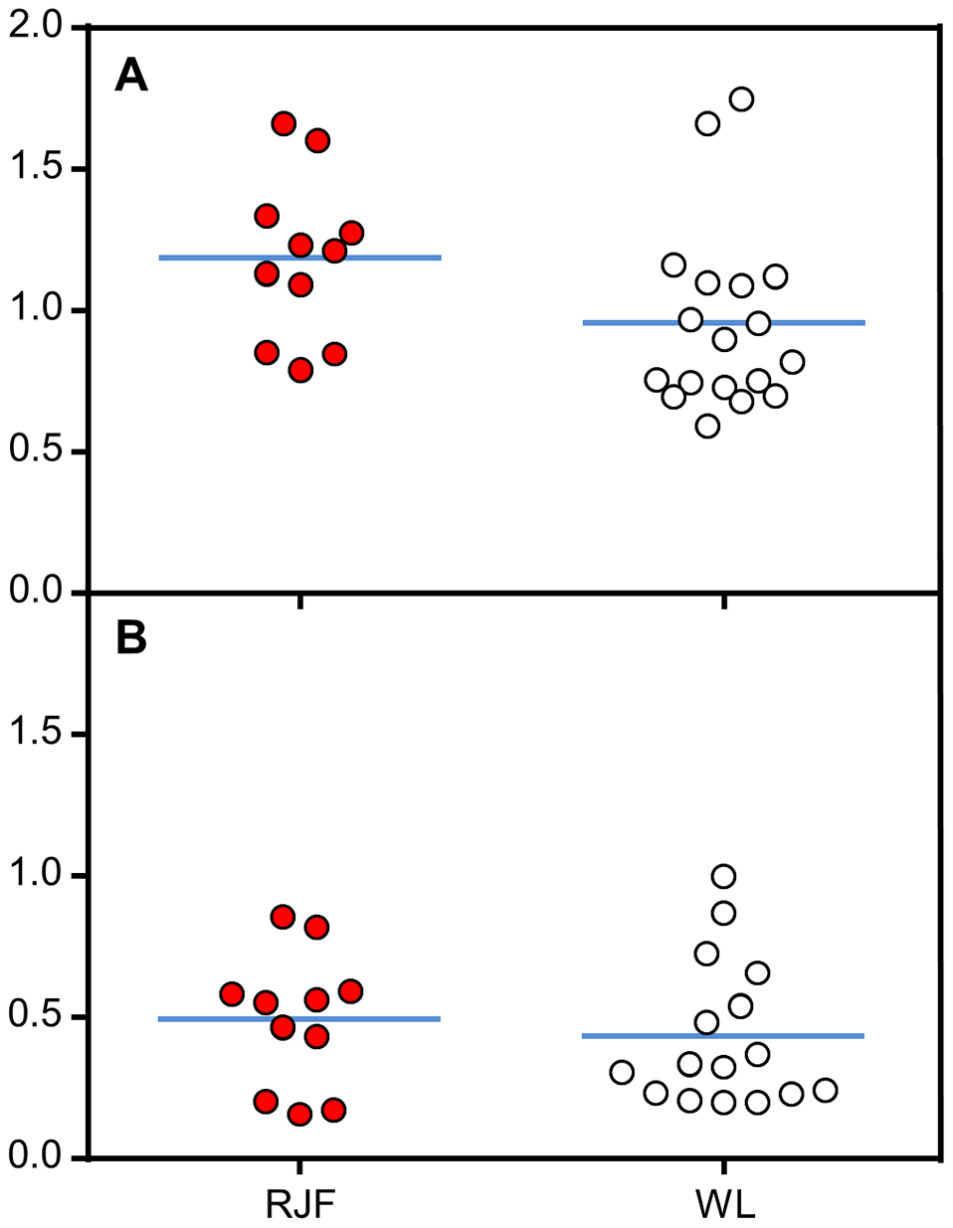
Adrenergic receptor gene expression in adrenal glands. A) *ADRA2C* and B) *ADRA2A* where the line indicates average value relative to housekeeping genes and the dots are the individual data points.

Because of very low ADRA2C receptor density in the adrenals, gene expression was analyzed instead. *ADRA2C* expression did not differ between the strains (Figure 6A). Compared to *ADRA2A* expression, *ADRA2C* was the predominantly expressed adrenergic receptor in the adrenals (Figure 6B). In the hypothalamus, there was no significant difference between RJF and WL chickens on *ADRA2C* expression (data not shown).

## Discussion

On chromosome 4 in the chicken genome there is a strong selective sweep showing signatures of selection in White leghorn chickens [12]. This area is void of genes except for ADRA2C, an autoregulatory transmembrane receptor of epinephrine and norepinephrine release in the CNS and the adrenal glands directly linked to the fight-and flight response and fearfulness [13]–[15]. The promoter region is hypermethylated in WL further highlighting it as an important gene in domestication [16]. The present study shows that despite the highly suggestive initial premises, ADRA2C is probably not involved in the domestication of stress-related phenotypes in WL chickens. No differences were found in expression levels in adrenals and hypothalamus, nor the amount of ADRA2C in relevant brain areas. It should be noted that the gene is downstream and outside of the sweep region, so any effect related to this gene would rely on, for example, an enhancer present within the sweep. Furthermore, behavior studies fail to show differences related to stress in an F_10_ advanced intercross and growth and fecundity remained unaltered.

The selective sweep on chromosome 4 is specific for layer breeds only, suggesting that this region is important for egg production. Indeed, earlier work indicates a connection between adrenergic control and fecundity [24] and Rubin et al (2010) suggest that the sweep is associated with egg production traits [12]. However, our measurements of egg production in terms of number of eggs laid and relative egg size in the F_10_ population did not support that hypothesis (Figure 2). Furthermore, an ongoing QTL analysis attempting to find regions demonstrating signatures of selection for fecundity has so far failed to find any QTL on chromosome 4 (pers. comm. D. Wright). In addition, earlier work on adrenergic control of egg production [24] failed to distinguish between adrenergic receptor subtypes and other subtypes may in fact be involved. Hence, ADRA2C does not seem to be associated with fecundity.

Low fearfulness and high stress thresholds must have been essential traits for animals during early domestication. ADRA2C is the main modulator of epinephrine secretion from the adrenals [14] and could therefore be a good target for domestication remodeling. Here we show that *ADRA2C* is expressed significantly more than *ADRA2A* in adrenals suggesting that ADRA2C, as in mice and rats, is the main controller of epinephrine release inhibition in the adrenals. However, no difference between RJF and WL on *ADRA2C* expression in the adrenals was found (Figure 6) and stress related behavior in the F_10_ population showed no differences between genotype in TI and OF (Figure 3 and 4). These findings do not support the hypothesis that domestication has altered adrenal ADRA2C phenotype. A small but significant difference was observed between A^+^/A^+^ and A^+^/A^D^ as well as A^D^/A^D^ in relaxed behavior after exposure to an aerial predator (Figure 4C–D). The absolute values of this behavior are altogether very low in all groups, ranging 0–5% of all expressed behaviors after exposure so the significant difference is probably of little biological relevance.

ADRA2C is expressed in the CNS, where it is co-operating with ADRA2A in a presynaptic negative feedback loop on norepinephrine release. ADRA2A controls high action potential stimulation frequencies, and ADRA2C does the same at low action potential stimulation frequencies [25] suggesting that a domestication difference on ADRA2C has a fine tuning effect. Fear of humans and surviving captivity should in early in domestication be the prime selection pressure shaping the domestic phenotype [7] and behavior studies are therefore suitable to detect the impact of ADRA2C on domestication. *Adra2C* is along with *Adra2A* strongly expressed in the amygdaloid complex, hypothalamus, olfactory system and the hippocampal formation in mice and rats (amygdala, hypothalamus and hippocampus are brain regions associated with stress responses). *Adra2C* is, in comparison to *Adra2A* significantly higher expressed in striatum [26],[27] where it is expressed in GABAergic medium spiny neurons (most common neuron in the striatum constituting of up to 95% of total neurons). These neurons participate in the regulation of mesencephalic dopaminergic neurons in substantia nigra [28] and *Adra2C* is expressed in these cells as well. Under input from striatum dopaminergic neurons are important for memory function [29]. Striatum does not have adrenergic neurons [28] suggesting that expression in these regions cannot be associated with stress or fear-related behaviors. Associative learning test showed no differences between genotypes, except male A^+^/A^+^ that solved the challenge significantly better than A^D^/A^D^ and A^+^/A^D^ (Figure 5). However, in biological terms the difference is again small. Receptor binding results in chicken demonstrates that ADRA2C, similar to mice and rat, is present in the amygdala, hypothalamus and striatum. This shows that it is present in brain regions associated with stress but also regions that are not. However, the level of ADRA2C was not influenced by *ADRA2C* genotype (Figure 5). *ADRA2C* expression in hypothalamus measured with qPCR showed no significant difference between RJF and WL.

Genomic methylation is a genetic modulator expected to be inheritable and robust [30]. The hypermethylation in the region of the *ADRA2C* promoter in WL compared to RJF indicate a modulation in expression, but the present study fails to demonstrate this. The methylation difference was shown to be robust between parents and offspring [16], and the study animals are no more than four generations apart from the present study cohort suggesting that the absence of expression difference is not due to loss of methylation patterns.

There is always a risk that a study, no matter the number of assays used, fails to demonstrate an effect due to tissue selectiveness, or that the assays were performed outside the temporal window of causation. The appropriate comparison was not executed in this study, but if the selective trait is so elusive, it is unlikely to have been important in this trait during the domestication of the chicken.

Our results indicate that the signature of selection on chromosome 4 is related to other gene elements than *ADRA2C*. Since no genes are known in the region it seems unlikely that the selective sweep is associated with the expression of a protein directly. Other genetic elements, such as miRNA or gene enhancers might be the selected targets. The expression of those may be influenced by transmodulatory elements lost by recombination in the F_10_ chickens. Theoretically, since the SNP used to genotype the animals in our study was outside the sweep region (within the gene), it would be possible that the regulatory regions were lost due to recombination. However, we further genotyped the inter-cross birds over the entire sweep region on a SNP 260 kb downstream from the SNP within ADRA2C, and found less than 65% recombinant individuals. A SNP 250 kb upstream from ADRA2C revealed only 4% recombinants. Although such recombination may in principle dilute the effects, it could not possibly explain the lack of expression differences in the parental birds.

This study demonstrates the need of cautiousness when interpreting results from whole genomic studies. It also highlights the necessity of these in depth studies, because good candidate genes in GWAS, QTL and selective sweep analysis are merely candidates until their causative effect is proven.

## Supporting Information

Table S1 Genomic coordinates for the different ADRA2 genes in different species used to generate the phylogenetic tree in Figure 1B. (TIF)

Table S2 Primer sequences used in the gene expression study and for genotyping F_10_ intercross birds. (TIF)

Table S3 Body mass from hatching to 88 days of age in F_10_ intercross birds separated for genotype: A^+^/A^+^ - homozygote for the RJF wild type allele, A^D^/A^D^ - homozygotes for the WL domestic allele, A^+^/A^D^ – heterozygote. n indicates the number of individuals for each genotype at each age. Data as mean (standard deviation). (TIF)

## References

[1] Price EO (2002) Animal domestication and behavior [electronic resource]. CABI

[2] Schütz K, Kerje S, Carlborg Ö, Jacobsson L, Andersson L, et al (2002) QTL Analysis of a Red Junglefowl×White Leghorn Intercross Reveals Trade-Off in Resource Allocation Between Behavior and Production Traits. Behavior Genetics 32: 423–433. 12467340 10.1023/a:1020880211144

[3] Schütz KE, Jensen P (2001) Effects of Resource Allocation on Behavioural Strategies: A Comparison of Red Junglefowl (*Gallus gallus*) and Two Domesticated Breeds of Poultry. Ethology 107: 753–765.

[4] Campler M, Jöngren M, Jensen P (2009) Fearfulness in red junglefowl and domesticated White Leghorn chickens. Behavioural Processes 81: 39–43. 19154782 10.1016/j.beproc.2008.12.018

[5] Johnsson M, Gustafson I, Rubin C-J, Sahlqvist A-S, Jonsson KB, et al (2012) A Sexual Ornament in Chickens Is Affected by Pleiotropic Alleles at *HAO1* and *BMP2*, Selected during Domestication. PLoS Genet 8: e1002914. 22956912 10.1371/journal.pgen.1002914 PMC3431302

[6] Eriksson J, Larson G, Gunnarsson U, Bed'hom B, Tixier-Boichard M, et al (2008) Identification of the *Yellow Skin* Gene Reveals a Hybrid Origin of the Domestic Chicken. PLoS Genet 4: e1000010. 18454198 10.1371/journal.pgen.1000010 PMC2265484

[7] Jensen P (2006) Domestication—From behaviour to genes and back again. Applied Animal Behaviour Science 97: 3–15.

[8] Andersson L (2012) How selective sweeps in domestic animals provide new insight into biological mechanisms. Journal of Internal Medicine 271: 1–14. 21895806 10.1111/j.1365-2796.2011.02450.x

[9] De Koning D, Haley C, Windsor D, Hocking P, Griffin H, et al (2004) Segregation of QTL for production traits in commercial meat-type chickens. Genetical Research 83: 211–220. 15462414 10.1017/s0016672304006846

[10] Schreiweis M, Hester P, Settar P, Moody D (2006) Identification of quantitative trait loci associated with egg quality, egg production, and body weight in an F2 resource population of chickens. Animal genetics 37: 106–112. 16573524 10.1111/j.1365-2052.2005.01394.x

[11] Wright D, Kerje S, Lundström K, Babol J, Schütz K, et al (2006) Quantitative trait loci analysis of egg and meat production traits in a red junglefowl×White Leghorn cross. Animal Genetics 37: 529–534. 17121597 10.1111/j.1365-2052.2006.01515.x

[12] Rubin C-J, Zody MC, Eriksson J, Meadows JRS, Sherwood E, et al (2010) Whole-genome resequencing reveals loci under selection during chicken domestication. Nature 464: 587–591. 20220755 10.1038/nature08832

[13] Philipp M, Hein L (2004) Adrenergic receptor knockout mice: distinct functions of 9 receptor subtypes. Pharmacology & Therapeutics 101: 65–74. 14729393 10.1016/j.pharmthera.2003.10.004

[14] Brede M, Nagy G, Philipp M, Sørensen JB, Lohse MJ, et al (2003) Differential Control of Adrenal and Sympathetic Catecholamine Release by α2-Adrenoceptor Subtypes. Molecular Endocrinology 17: 1640–1646. 12764077 10.1210/me.2003-0035

[15] Sallinen J, Haapalinna A, Viitamaa T, Kobilka BK, Scheinin M (1998) Adrenergic α2C-Receptors Modulate the Acoustic Startle Reflex, Prepulse Inhibition, and Aggression in Mice. The Journal of Neuroscience 18: 3035–3042. 9526020 10.1523/JNEUROSCI.18-08-03035.1998 PMC6792602

[16] Natt D, Rubin C-J, Wright D, Johnsson M, Belteky J, et al (2012) Heritable genome-wide variation of gene expression and promoter methylation between wild and domesticated chickens. BMC Genomics 13: 59. 22305654 10.1186/1471-2164-13-59 PMC3297523

[17] Richards EJ (2006) Inherited epigenetic variation—revisiting soft inheritance. Nature Reviews Genetics 7: 395–401. 10.1038/nrg1834 16534512

[18] Wright D, Rubin CJ, Martinez Barrio A, Schütz K, Kerje S, et al (2010) The genetic architecture of domestication in the chicken: effects of pleiotropy and linkage. Molecular Ecology 19: 5140–5156. 21040053 10.1111/j.1365-294X.2010.04882.x

[19] Forkman B, Boissy A, Meunier-Salaün MC, Canali E, Jones RB (2007) A critical review of fear tests used on cattle, pigs, sheep, poultry and horses. Physiology & Behavior 92: 340–374. 18046784 10.1016/j.physbeh.2007.03.016

[20] Agnvall B, Jöngren M, Strandberg E, Jensen P (2012) Heritability and genetic correlations of fear-related behaviour in red Junglefowl–possible implications for early domestication. PloS one 7: e35162. 22536354 10.1371/journal.pone.0035162 PMC3334967

[21] Håkansson J, Jensen P (2008) A longitudinal study of antipredator behaviour in four successive generations of two populations of captive red junglefowl. Applied Animal Behaviour Science 114: 409–418.

[22] Livak KJ, Schmittgen TD (2001) Analysis of Relative Gene Expression Data Using Real-Time Quantitative PCR and the 2−ΔΔCT Method. Methods 25: 402–408. 11846609 10.1006/meth.2001.1262

[23] Drummond GB, Vowler SL (2012) Different tests for a difference: how do we do research? Microcirculation 19: 188–191. 22329830 10.1111/j.1549-8719.2012.00162.x

[24] Moudgal RP, Razdan MN (1981) Induction of ovulation in vitro by LH and catecholamines in hens is mediated by α-adrenergic receptors. Nature 293: 738–739. 6270572 10.1038/293738a0

[25] Hein L, Altman JD, Kobilka BK (1999) Two functionally distinct α2-adrenergic receptors regulate sympathetic neurotransmission. Nature 402: 181–184. 10647009 10.1038/46040

[26] Nicholas AP, Pieribone V, Hökfelt T (1993) Distributions of mRNAs for alpha-2 adrenergic receptor subtypes in rat brain: An in situ hybridization study. The Journal of Comparative Neurology 328: 575–594. 8381444 10.1002/cne.903280409

[27] Wang R, Macmillan LB, Fremeau RT Jr, Magnuson MA, Lindner J, et al (1996) Expression of α2-adrenergic receptor subtypes in the mouse brain: evaluation of spatial and temporal information imparted by 3 kb of 5′ regulatory sequence for the α2A AR-receptor gene in transgenic animals. Neuroscience 74: 199–218. 8843087 10.1016/0306-4522(96)00116-9

[28] Holmberg M, Scheinin M, Kurose H, Miettinen R (1999) Adrenergic α2C-receptors reside in rat striatal GABAergic projection neurons: comparison of radioligand binding and immunohistochemistry. Neuroscience 93: 1323–1333. 10501456 10.1016/s0306-4522(99)00260-2

[29] Rosin DL, Talley EM, Lee A, Stornetta RL, Gaylinn BD, et al (1996) Distribution of α2C-adrenergic receptor-like immunoreactivity in the rat central nervous system. The Journal of Comparative Neurology 372: 135–165. 8841925 10.1002/(SICI)1096-9861(19960812)372:1<135::AID-CNE9>3.0.CO;2-4

[30] Li Y, O'Neill C (2012) Persistence of Cytosine Methylation of DNA following Fertilisation in the Mouse. PLoS ONE 7: e30687. 22292019 10.1371/journal.pone.0030687 PMC3266909

